# Glial Modulation by *N*-acylethanolamides in Brain Injury and Neurodegeneration

**DOI:** 10.3389/fnagi.2016.00081

**Published:** 2016-04-26

**Authors:** María I. Herrera, Rodolfo Kölliker-Frers, George Barreto, Eduardo Blanco, Francisco Capani

**Affiliations:** ^1^Instituto de Investigaciones Cardiológicas, Facultad de Medicina, Universidad de Buenos Aires – Consejo Nacional de Investigaciones Científicas y TécnicasBuenos Aires, Argentina; ^2^Centro de Investigaciones en Psicología y Psicopedagogía, Facultad de Psicología, Universidad Católica ArgentinaBuenos Aires, Argentina; ^3^Department of Nutrition and Biochemistry, Faculty of Sciences, Pontificia Universidad JaverianaBogotá, Colombia; ^4^Departament de Pedagogia i Psicologia, Facultat d’Educació, Psicologia i Treball Social, Universitat de LleidaLleida, Spain; ^5^Facultad de Psicología, Universidad Católica ArgentinaBuenos Aires, Argentina; ^6^Departamento de Biología, Universidad Argentina John F. KennedyBuenos Aires, Argentina; ^7^Instituto de Ciencias Biomédicas, Facultad de Ciencias de la Salud, Universidad Autónoma de ChileSantiago, Chile

**Keywords:** neuroinflammation, gliosis, *N*-acylethanolamides, PPARα, neuroprotection

## Abstract

Neuroinflammation involves the activation of glial cells and represents a key element in normal aging and pathophysiology of brain damage. *N*-acylethanolamides (NAEs), naturally occurring amides, are known for their pro-homeostatic effects. An increase in NAEs has been reported *in vivo* and *in vitro* in the aging brain and in brain injury. Treatment with NAEs may promote neuroprotection and exert anti-inflammatory actions via PPARα activation and/or by counteracting gliosis. This review aims to provide an overview of endogenous and exogenous properties of NAEs in neuroinflammation and to discuss their interaction with glial cells.

## Introduction

Brain injury includes several conditions that damage the brain and alter its function, such as trauma, stroke, hypoxia, and infection. Although this term generally refers to sudden insults, neurodegenerative disorders are also sources of brain damage (Lukersmith et al., 2016). There are still no effective treatments for most cases of brain injury and numerous gaps regarding their physiopathology. However, research has elucidated the possible detrimental effects of the inflammatory response mediated by glial cells. If this response is uncontrolled and chronic, it may lead to neurotoxicity (Arevalo et al., 2012) and disruption of endogenous neurogenesis (Whitney et al., 2009). This phenomenon known as *neuroinflammation* is also evident in the aging brain, which is particularly vulnerable to neurodegenerative and neuropsychiatric disease (Matt and Johnson, 2016; Mohan et al., 2016). Therefore, neuroinflammation is a key target for neuroprotection and repair (Skaper et al., 2013, 2014), which constitutes a promising alternative for brain injury and neurological disorders.

*N*-acylethanolamides (NAEs) are endogenous lipid mediators which apparently elicit a pro-homeostatic role in response to neuroinflammation (Esposito and Cuzzocrea, 2013; Skaper et al., 2013). In addition, exogenous administration of NAEs could exert neuroprotective effects by reducing neuroinflammation, as it has been demonstrated in several models of β-amyloid- induced astrogliosis (Scuderi et al., 2011, 2012; Benito et al., 2012), Alzheimer disease (D’Agostino et al., 2012; Scuderi et al., 2014), Parkinson disease (Galan-Rodríguez et al., 2009; Esposito et al., 2012; González-Aparicio et al., 2014), stroke (Ahmad et al., 2012b), cerebral ischemia (Zhou et al., 2012), traumatic brain injury (Ahmad et al., 2012a), spinal cord injury (SCI) (Genovese et al., 2008; Esposito et al., 2011), peripheral neuropathy (Mannelli et al., 2013) and lipopolysaccharide (LPS)-induced neuroinflammation (Sayd et al., 2015). The aim of this review is to provide an overview of recent progress related to the endogenous and exogenous neuroprotective and anti-inflammatory properties of NAEs, emphasizing their effects on glial cells.

## Astrocytes, Microglia, and Neuroinflammation

Astrocytes contribute to maintain homeostasis in the central nervous system (CNS) by providing nutrients to neurons, recycling neurotransmitters and regulating synaptic plasticity (Pekny and Pekna, 2015). Astrocytes also have a critical role in the regulation of neural immune response and neuronal survival, promoting wound healing and tissue repair upon CNS insults (Wang and Bordey, 2008; Jha et al., 2016). Modifications in gene expression, hypertrophy and proliferation of astrocytes are involved in preserving neuronal function (Sofroniew, 2005). Microglia also responds to brain injury. It constitutes the resident macrophage population of the CNS (Hanisch and Kettenmann, 2007; Matt and Johnson, 2016) that migrates to the injury site and initiates communication with the immune system (Robel et al., 2011). While astrocytes monitor extracellular fluid, pH and ion homeostasis in order to promote the recovery of injured tissue, microglia scavenges dead cells, and secretes neurotrophic factors. This process is known as *reactive gliosis* (Skaper et al., 2014), and is characterized by molecular, morphological and functional changes in glial phenotype in response to brain injury (Zamanian et al., 2012; Pekny and Pekna, 2015).

Cytokines mediate communication between nervous and immune system (Skaper et al., 2013, 2014), which is known as *neuroimmunomodulation* (Arevalo et al., 2012). Therefore, besides generating pro-inflammatory molecules, glial cells respond to pro-inflammatory signals released from mast cells (Skaper and Facci, 2012), and cytokines recruit glia to the focus of inflammation (Dong and Benveniste, 2001; Rebenko-Moll et al., 2006; Rostène et al., 2007).

However, if glial activation is excessive, the inflammatory response oriented to protect neural tissue might override the bounds of physiological control (Skaper et al., 2013, 2014) and reactive gliosis becomes dysfunctional (Pekny and Pekna, 2015). This phenomenum called *neuroinflammation* leads to neurotoxicity and promotes further injury (Arevalo et al., 2012). While acute insults are transient and rarely detrimental to neuronal survival, neuroinflammation is a chronic and self-perpetuating response, which may constitute a point of origin for neurological disorders (Jha et al., 2016). Non-resolving inflammation is a major cause of disease since inflammation can damage tissue and necrosis can exacerbate inflammation (Nathan and Ding, 2010). Moreover, uncontrolled inflammation promotes disruption of endogenous neurogenesis (Whitney et al., 2009) and synaptic dysfunction (Chung et al., 2015), inhibiting adaptive plasticity mechanisms needed for functional recovery (Pekny and Pekna, 2015).

Genes mediating neuroinflammation and immune system activation show significant age-related upregulation (Mohan et al., 2016). Aging acts as a *silent contributor* to neuroinflammation, establishing the condition as a central pathophysiological mechanism, maintaining, and impairing it (Sandu et al., 2015). Reactive gliosis and neuroinflammation may be triggered by the accumulation of proteins with abnormal conformations (e.g., β-amyloid) or by signals emanating from brain injury processes (e.g., hypoperfusion of neural tissue) in the aging brain (Gouw et al., 2011; Scuderi et al., 2011).

The aging brain is characterized by a sensitization to neuroinflammatory responses, which provokes abnormalities in brain structure and metabolism (Rosano et al., 2012). Aged microglia is primed to be activated and resistant to endogenous regulatory systems (Norden and Godbout, 2013). Hence, the response of microglia to stimulus involves a more robust and persistent production of pro-inflammatory cytokines, which compromises normal neuronal functionality (Boche et al., 2013; Perry and Teeling, 2013). Microglia develops a loss of integrated regulatory networks. Therefore, homeostasis in brain-immune interactions is considerably altered and reduced. This neuro-immune dysfunction is associated with a low-grade chronic neuroinflammation, which contributes to cognitive deficits and susceptibility to age-related pathologies (Matt and Johnson, 2016): neurodegenerative and neuropsychiatric diseases (Mohan et al., 2016).

Neuroinflammation represents a key element in brain aging (Matt and Johnson, 2016; Mohan et al., 2016) and in the pathophysiology of several neurological diseases (Skaper et al., 2014, 2015): neurodegenerative disorders (Giovannini et al., 2002; Dauer and Przedborski, 2003; McGeer and McGeer, 2013; Amor et al., 2014), stroke (Iadecola and Anrather, 2011), chronic neuropathic pain (Myer et al., 2006; Tenorio et al., 2013), among others. Therefore, neuroinflammation constitutes an important target for neuroprotection (Skaper et al., 2013, 2014). Although mechanisms of neuroinflammation are probably similar in aging and a wide range of neurological diseases, these conditions differ in etiology and in how the inflammatory response contributes to progressive damage. A thorough understanding of these molecular pathways is necessary for designing neuroprotective strategies (Sandu et al., 2015).

## The *N*-acylethanolamides Signaling System

**FIGURE 1:**
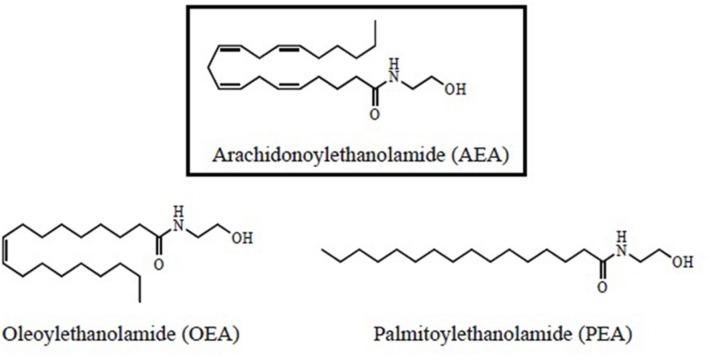
**Structural formulas for *N*-acylethanolamides (NAEs)**.

**FIGURE 2:**
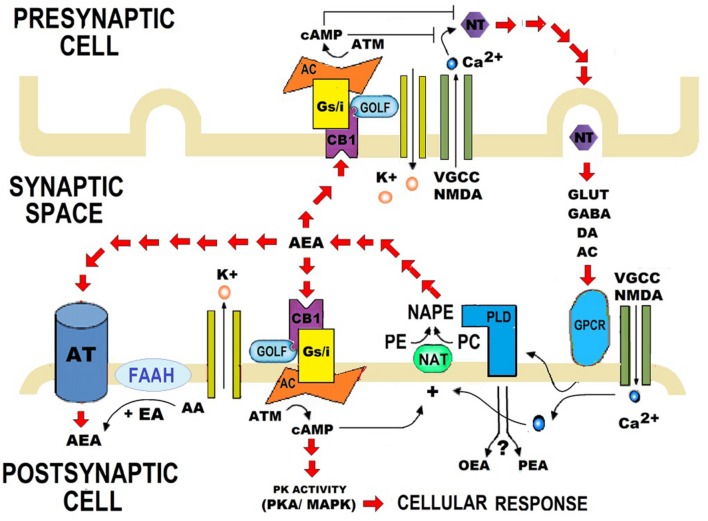
**Role of synthesis and catalysis of *N*-acylethanolamides (NAEs) on the synaptic transmission.** Anandamide (AEA) is synthesized when *N*-acylphosphatidyletanolamide (NAPE) is hydrolysed by a specific phospholipase D (PLD*)*. AEA is also synthesized from arachidonic acid (AA) and ethanolamide. When AA is replaced by palmitic acid (PA) or oleic acid (OA), Palmitoylethanolamide (PEA), or Oleoylethanolamide (OEA) are synthesized respectively. PA and OA are AA conjugates with other compounds, and therefore PEA and OEA are considered as AEA derivatives. AEA contributes to bioelectrical plasticity, by acting as a retrograde, or anterograde messenger. After performing in CB1 and CB2 receptors, AEA is catalyzed by fatty acid amidohydrolase (FAAH). FAAH is the primary catabolic enzyme for AEA, and it also regulates the contents of PEA and OEA, which do not activate CB1 and CB2. Instead, PEA and OEA apparently activate non-cannabinoid receptors, such as PPARα. Nevertheless, the role of PEA and OEA on synapsis is still unclear. NT, Neurotransmitter; DA, Dopamine; AC, Acetylcholine; VGCC, Voltage-gated calcium channel; NMDA, *N*-Methyl-D-aspartate; GPCR, G protein–coupled receptors; PE, Phosphatidyl ethanolamine; PC, Phosphatidylcholine; NAT, *N*-acetyltransferase; K^+^, Pottasium ion; Ca^2+^, Calcium ions; cAMP, Cyclic adenosine monophosphate; ATP, Adenosine triphosphate; AC, Adenylate cyclase; GOLF, Olfactory neuron specific-G protein involved in odorant signal transduction; Gs/i, Stimulating/Inhibitory G Protein; PK activity, Protein Kinase activity; AT, Anandamide Translocase.

*N*-acylethanolamides are present in human and murine brain in considerable amounts (Di Marzo, 1998; Maccarrone and Finazzi-Agró, 2002, 2003). NAEs are endogenous lipid mediators that include the endocannabinoid *N-arachidonoylethanolamide (anandamide or AEA)* and its congeners, the non-cannabimimmetic compounds *Palmitoylethanolamide (PEA)* and *Oleoylethanolamide (OEA)* (Skaper et al., 2013; Sayd et al., 2015) (**Figure 1**). AEA is synthesized by the hydrolysis of *N-acylphosphatidyletanolamide (NAPE)*, through the action of the enzyme *N-acylphosphatidylethanolamide-phospholipase D (NAPE-PLD)*. After performing in CB1 and CB2 receptors, AEA is degraded by the action of the enzyme *fatty acid amidohydrolase (FAAH).* AEA is also synthesized from ethanolamide and a fatty acid (arachidonic acid) (**Figure 2**). AEA is a transient signal synthesized on demand, from changes in cell membrane induced by stimulating glutamatergic, GABAergic, cholinergic, or dopaminergic receptors. This transient signal can act as a retrograde or anterograde messenger, either by inhibiting neurotransmitter release on presynaptic CB1 or by controlling postsynaptic depolarization (Piomelli, 2003; Rodríguez de Fonseca, 2012) (**Figure 2**).

*N*-acylethanolamides share biosynthetic and degradative mechanisms. Like AEA, PEA, and OEA are produced on demand through NAPE-PLD and catalyzed by FAAH (Rodríguez de Fonseca et al., 2001; Piomelli, 2003; Skaper et al., 2013; Sayd et al., 2015). PEA and OEA can be also synthesized from ethanolamine and a fatty acid: palmitic acid or oleic acid, respectively (Rodríguez de Fonseca, 2012; Scuderi et al., 2014) (**Figure 2**). Although PEA and OEA do not bind cannabinoid receptors (CB1 and CB2) (Rodríguez de Fonseca et al., 2001; Mackie and Stella, 2006; Scuderi et al., 2014; Vacondio et al., 2015), they can act as *entourage compounds*, enhancing the activity of AEA (De Petrocellis et al., 2001; Cravatt and Lichtman, 2002; Ross, 2003; Bradshaw and Walker, 2005; Re et al., 2007; Liput et al., 2014).

The biological effects of PEA and OEA are mainly mediated via the activation of the nuclear *Peroxisome Proliferator-Activated Receptor-alpha (PPARα)* (Rodríguez de Fonseca et al., 2001; Lo Verme et al., 2005; Sun et al., 2007). However, PEA and OEA might act through alternative receptors: the *transient receptor potential vaniloid type-1 (TRPV1)* (Van Der Stelt and Di Marzo, 2004; Wang et al., 2005; Almási et al., 2008; Thabuis et al., 2008), the *G protein-coupled receptors GPR55* and *GPR119* (Overton et al., 2006; Godlewski et al., 2009), and other peroxisome proliferator-activated receptor (PPAR) isoforms (*PPAR-δ and PPAR-γ*) (Fu et al., 2003; Paterniti et al., 2013). Although PEA and OEA are known to exert a protective role in response to brain injury and neuroinflammation (Esposito and Cuzzocrea, 2013), their specific role on synapsis is still unknown (**Figure 2**).

### Endogenous Pro-homeostatic Properties of *N*-acylethanolamides

Glial cells possess endogenous homeostatic molecules, such as NAEs, which can be up-regulated in response to harmful stimuli provoking inflammation (Skaper et al., 2013). *In vitro* studies have demonstrated that NAEs are produced by astrocytes (Walter et al., 2002) and PEA is synthesized and hydrolysed by microglia (Muccioli and Stella, 2008). NAEs are known to be accumulated in mammalian tissues as a result of membrane changes associated with necrosis (Berdyshev et al., 2000). Pronounced increases in NAEs were reported in glutamate-induced excitotoxicity in cultured cortical neurons (Hansen et al., 1995, 1997). These homeostatic signals regulate cell survival upon brain damage and neuroinflammation (Esposito and Cuzzocrea, 2013).

A release of OEA (up to 242 pmol/mL), PEA (up to 120 pmol/mL), and AEA (up to 42 pmol/mL) was reported *in vivo* during human stroke, suggesting the contribution of NAEs signaling system to downstream events in the ischemic cascade (Schäbitz et al., 2002). A significant accumulation of NAEs has been also observed in a murine model of stroke. Striatal and cortical NAEs concentrations were about 30-fold higher in the infarcted than in the non-infarcted hemisphere (Berger et al., 2004). In addition, an augmentation in PEA levels was observed in a murine model of focal cerebral ischemia (Franklin et al., 2003). Moreover, an accumulation of *N-acetylethanolamide phospholipids (NAPEs)* has been registered in rat brain during post-decapitative ischemia. NAPEs are precursors for NAEs and can be formed as a stress response during neuronal injury (Natarajan et al., 1986). This ischemia-induced synthesis is apparently age-dependent and more pronounced in developing than in adult brain (Moesgaard et al., 2000).

*N*-acylethanolamides might also exert a pro-homeostatic role in response to reactive gliosis and neuroinflammation in the aging brain. Recent *in vivo* studies have reported increased availability of AEA (Pascual et al., 2014) and NAPE-PLD (Di Marzo et al., 2015) during aging. PEA and OEA are up-regulated in activated cultured primary rat astrocytes after β*-amyloid (A*β*)* accumulation (Scuderi et al., 2011), a frequent event in the aging brain (Gouw et al., 2011). In addition, *in vitro* studies have also shown an increase of PEA levels in response to neurodegeneration and reactive gliosis in *organotypic hippocampal slice cultures (OHSCs)* (Kallendrusch et al., 2010).

Endogenous synthesis of NAEs might be an adaptive response for the down-modulation of mast cells hyperactivity and consequent expression of pro-inflammatory molecules (Esposito and Cuzzocrea, 2013). This mechanism is known by the *ALIA* acronym (*Autacoid Local Inflammation Antagonism)* (Aloe et al., 1993). Mast cells, non-neural and immune-related cells, are capable of crossing both compromised blood-spinal cord and blood–brain barrier in cases of CNS pathology. However, NAEs could also protect neurons against glutamate excitotoxicity (Skaper et al., 1996). This finding contributed to the understanding of the pro-homeostatic role of NAEs, which represents a broader local anti-injury function (*Autacoid Local Injury Antagonism*), not limited to an autacoid reduction of inflammation. These *ALIAmides* down-regulate mast cells, protect neurons against excitotoxicity, and inhibit the spread of necrosis, thus preventing secondary neuronal damage (Esposito and Cuzzocrea).

The *entourage hypothesis* was proposed in order to explain a possible mechanism of PEA and OEA pro-homeostatic properties. This hypothesis suggests that OEA and PEA inhibit the degradation of AEA by competing with AEA for FAAH catalytic activity, thus increasing AEA levels and cytoprotective action (De Petrocellis et al., 2001; Cravatt and Lichtman, 2002; Ross, 2003; Bradshaw and Walker, 2005; Re et al., 2007; Liput et al., 2014). For instance, increases of PEA levels were reported in early stages of SCI. This augmentation could be explained by the entourage hypothesis: an increase in AEA levels and down-regulation of FAAH was also registered (Garcia-Ovejero et al., 2009). However, another mechanism was proposed for the protective role of endogenous OEA and PEA: the activation of a nuclear receptor such as PPARα (Esposito and Cuzzocrea, 2013). The up-regulation of PEA and OEA in activated cultured primary rat astrocytes after Aβ accumulation elicited protective effects through PPARα activation, possibly to antagonize the effects of PPARα down-regulation caused by Aβ exposure. In fact, down-modulation of PPARα may represent one of the molecular mechanisms by which Aβ exerts its toxicity (Scuderi et al., 2011).

## Therapeutic Activity of *N*-acylethanolamides

Exogenous NAEs act apparently through *receptor pleiotropism*, i.e., interactions with different receptor targets, such as PPARα (Skaper et al., 2013). PPARs are known to regulate the expression of large gene arrays, thus modulating important metabolic events (Berger et al., 2005), innate and adaptive immunity (Daynes and Jones, 2002). *In vivo* studies have reported that the anti-inflammatory properties of PEA (Lo Verme et al., 2005) and OEA (Sun et al., 2007) associated with neuronal protection are exerted by activating PPARα. This anti-inflammatory effect observed in wild-type mice was absent in mutant PPARα-deficient mice (Lo Verme et al., 2005; Sun et al., 2007). PPARα-mediated neuroprotection was later replicated in several models of brain injury and neurodegeneration. Another mechanism associated with NAEs therapeutic action is down-regulation of glial activity, as it is described below and summarized in **Table 1**.

**Table 1:** Therapeutic effects of *N*-acylethanolamides (NAEs) in several *in vivo* and *in vitro* models of brain damage.

Model of brain damage	Therapeutic action of OEA, PEA, or AEA	Reference
Aβ-induced astrogliosis (*In vitro*)	Reduction in TNFα, COX-2, and iNOS expression	Benito et al., 2012

**Model of brain damage**	**Therapeutic action of OEA**	**Reference**

Acute cerebral ischemia (Middle cerebral artery occlusion in mice)	Dose-dependent reduction of infarct volume and brain edema	Zhou et al., 2012
	Dose-dependent improvement in neurological dysfunction	
	PPARα activation	
Perinatal asphyxia (PA) (Water bath at 37°C for 20 min immediately after delivery)	Improvement in exploratory locomotion at postnatal day 30	Herrera et al., 2014
Parkinson Disease (PD) (6-OHDA-induced striatal lesion in rats)	Dose-dependent reduction in reactive microglia activation (OX6 expression) and oxidative response (HO-1 expression)	Galan-Rodríguez et al., 2009
6-OHDA induced degeneration of substantia nigra dopamine neurons *(In vitro)*	Dose-dependent protection against toxicity and cell death	Galan-Rodríguez et al., 2009
Parkinson Disease (6-OHDA-induced striatal lesion in rats)	Dose-dependent reduction in neurotoxicity and oxidative response (HO-1 expression)	González-Aparicio et al., 2014
	Dose-dependent reduction of behavioral deficits	
6-OHDA induced degeneration of substantia nigra dopamine neurons *(In vitro)*	Dose-dependent protection against 6-OHDA-induced toxicity PPARα activation	González-Aparicio et al., 2014
Lipopolysaccharide-induced neuroinflammation (LPS injection in rats)	Enhancement of the hypothermic response after LPS injection	Sayd et al., 2015
	Reduction in oxidative/nitrosative stress, IL-1β, COX-2, PGE2, and TNF-α mRNA expression	
	Disruption of anhedonia	

**Model of brain damage**	**Therapeutic action of PEA**	**Reference**

Lipopolysaccharide-induced neuroinflammation (LPS injection in rats)	Enhancement of the hypothermic response after LPS injection	Sayd et al., 2015
	Reduction in oxidative/nitrosative stress, IL-1β, COX-2, and PGE2 expression	
Stroke (Middle cerebral artery occlusion in rats)	Reduction of edema, brain infarction, and lesion size	Ahmad et al., 2012b
	Improvement in apoptosis level	
	Blockage of astrocytes infiltration	
	Reduction of motor deficits	
Traumatic brain injury (TBI) [Controlled cortical impact (CCI) in mice]	Reduction of lesion size	Ahmad et al., 2012a
	Improvement in apoptosis level	
	Blockage of astrocytes infiltration	
	Improvement in neurobehavioral functions	
Spinal cord injury (SCI) (Compression model in mice)	Reduction of tissue injury	Genovese et al., 2008
	Reduction in the degree of apoptosis and pro-inflammatory cytokine expression	
	Recovery of motor limb function	
	PPARα activation	
Spinal cord injury (Compression model in mice)	Reduction in mast cell infiltration and activation	Esposito et al., 2011
	Reduction in activation of microglia and astrocytes expressing CB2	
	Changes in the expression of neurotrophic factors and in spinal cord dopaminergic function	
Peripheral neuropathy [Chronic constriction injury (CCI) in mice]	Reduction of edema	Mannelli et al., 2013
	Reduction in macrophage infiltration	
	Augmentation in myelin sheath and axonal diameter	
	Anti-nociception	
	PPARα activation	
Alzheimer Disease (AD) (Aβ peptide injection in mice)	Reduction in lipid peroxidation, protein nytrosylation, iNOS induction, and caspase 3 activation expression	D’Agostino et al., 2012
	Dose-dependent reduction or prevention of learning and memory impairment	
	PPARα activation	
Alzheimer Disease (Aβ peptide injection in rats)	Reversion of reactive gliosis, amyloidogenesis, and tau protein hyperphosphorylation	Scuderi et al., 2014
	Reduction of mnestic deficits	
	PPARα activation	
Aβ-induced astrogliosis (*In vitro*)	Reduction in astrocytes activation	Scuderi et al., 2012
	Improvement in neuronal survival	
	PPARα activation	
Aβ-induced astrogliosis (*In vitro*)	Reduction in iNOS, COX-2, nitric oxide, IL-1β, TNF-α, and PGE2 expression	Scuderi et al., 2011
	PPARα activation	
Parkinson Disease (MPTP injection in mice)	Protection against neurotoxicity and the ensuing functional deficits, loss of TH positive neurons, and alterations of microtubule-associated protein 2a,b, dopamine transporter and nNos-positive cells	Esposito et al., 2012
	Reduction in microglial activation and the number of GFAP-positive astrocytes	
	Reversion of motor deficits	
	PPARα activation	


### Neuroprotection in Experimental Models of Brain Injury

Oral OEA pre-treatment (10, 20, and 40 mg/kg, for 3 days before ischemia) could exert neuroprotective effects against acute cerebral ischemic injury in mice. Transient focal cerebral ischemia was induced by middle cerebral artery occlusion (MCAo) for 90 min followed by reperfusion. OEA pre-treatment was associated with an improvement in neurological dysfunction and a reduction in infarct volume and brain edema. This effect was exerted via PPARα in a dose-dependent manner with 40 mg/kg as the most effective dose (Zhou et al., 2012). In addition, administration of OEA (10 mg/kg) in rats subjected to perinatal asphyxia (PA) was associated with an improvement in exploratory locomotion at postnatal day 30. PA was induced by a water bath at 37°C for 20 min immediately after delivery. Currently, further research is being developed in order to study the morphological effects of OEA on PA (Herrera et al., 2014). Moreover, neuroprotective properties of PEA were reported in a rat model of stroke through MCAo. PEA treatment (10 mg/kg, 1 h after ischemia and 6 h after reperfusion) reduced edema, brain infarction, and lesion size, and improved apoptosis level (assayed by Bax and Bcl-2). Infiltration of astrocytes was blocked and motor deficits were reduced (Ahmad et al., 2012b). Similar results were reported after PEA treatment in mice subjected to an experimental model of *traumatic brain injury (TBI). Controlled cortical impact (CCI)* was performed in adult mice and produced full thickness lesions in sensorimotor cortex. PEA treatment (10 mg/kg, 1 h after TBI) could ameliorate secondary damage by reducing lesion size and blocking infiltration of astrocytes. Moreover, apoptosis level and neurobehavioral functions were improved. (Ahmad et al., 2012a).

The effect of PEA on secondary damage induced by SCI was also tested. SCI was induced in mice by application of vascular clips to the dura mater via a four-level T5–T8 laminectomy (*compression model*). Repeated PEA administration (10 mg/kg, 30 min before, 1 and 6 h after SCI) could significantly ameliorate the recovery of motor limb function and reduce the degree of tissue injury and inflammation, pro-inflammatory cytokine expression, apoptosis, among other biomarkers. This protective action was exerted through PPARα activation (Genovese et al., 2008). Further evidence supported the neuroprotective properties of PEA in SCI. SCI was induced by applying an aneurysm clip to the spinal cord in mice, thus replicating the persistence of cord compression (compression model). PEA treatment (10 mg/kg, 6 and 12 h after SCI) was able to diminish mast cell infiltration and reduce the activation of microglia and astrocytes expressing CB2 receptor after SCI. These modifications were accompanied by changes in the expression of neurotrophic factors and in spinal cord dopaminergic function (Esposito et al., 2011).

Neuroprotective effects of PEA were observed in a murine model of peripheral neuropathy, which induces damage of the sciatic nerve by *chronic constriction injury (CCI).* Peripheral nerve was rescued from inflammation and structural derangement after repeated daily treatments with PEA (30 mg/kg). A reduction of edema and macrophage infiltration, and an augmentation in myelin sheath and axonal diameter were observed. In addition, anti-nociceptive effects were registered. These changes apparently occurred by a PPARα-mediated mechanism since they were absent in PPARα null mice (Mannelli et al., 2013).

Finally, neuroprotective properties of OEA (10 mg/kg) and PEA (10 mg/kg) were recently tested in a murine model of *LPS*-induced neuroinflammation. Both pre-treatments with OEA and PEA (10 min before LPS administration) could potentiate the hypothermic response after LPS injection, ameliorate LPS-induced oxidative/nitrosative stress, and reduce the expression of *interleukin-1*β *(IL-1*β*)*, *cyclooxygenase-2 (COX-2)*, and *prostaglandin E_2_ (PGE2)*. However, only OEA was able to reduce brain *tumor necrosis factor-α (TNF-α)* mRNA and disrupt LPS-induced anhedonia in a saccharine preference test (Sayd et al., 2015).

### Neuroprotection in Experimental Models of Neurodegeneration

Exogenous NAEs have exerted neuroprotective activities in experimental models of *Alzheimer disease (AD).* PEA reduced (10 mg/kg) or prevented (30 mg/kg) learning and memory impairment in mice injected intracerebroventricullarly with Aβ25–35 peptide (9 nmol). This therapeutic possibility to treat memory deficits associated with AD was apparently mediated by activation of PPARα. Besides behavioral improvement, PEA treatment (once a day, starting 3 h after Aβ25–35, for 1 or 2 weeks) reduced the expression of experimental molecular and biochemical markers induced by Aβ25–35: lipid peroxidation, protein nytrosylation, *inducible nitric oxide synthase (iNOS)* induction and caspase 3 activation (D’Agostino et al., 2012). PEA could exert anti-inflammatory and neuroprotective effects in another experimental model of AD. Adult male rats were injected intrahippocampally with beta Aβ1–42. PEA treatment (10 mg/kg, once a day for 7 consecutive days, starting from the day of the surgery) reduced mnestic deficits and restored reactive gliosis, amyloidogenesis and tau protein hyperphosphorylation through PPARα involvement. These results suggested that PEA could have potential to alleviate the cognitive symptoms and to modify disease progression (Scuderi et al., 2014).

Palmitoylethanolamide treatment results in a decreased Aβ-induced astrocyte activation and improves neuronal survival via PPARα. Primary rat mixed neuroglial co-cultures and organotypic hippocampal slices were challenged with Aβ1–42 and treated with PEA. The findings of this study reveal the neuroprotective effect of reactive gliosis reduction (Scuderi et al., 2012). Similar *in vitro* studies from the same laboratory were aimed at assessing the effect of exogenous PEA on the production of pro-inflammatory molecules induced by Aβ: iNOS, COX-2, nitric oxide, IL-1β, TNF-α, and PGE2. PEA could blunt the expression and release of all the pro-inflammatory factors. These effects were attenuated by a PPARα antagonist, suggesting that the anti-inflammatory properties of PEA may be mediated by this nuclear receptor (Scuderi et al., 2011). Moreover, exogenous OEA, PEA, and AEA have shown an anti-inflammatory response *in vitro* by preventing the increase in TNFα, COX-2, and iNOS induced by the pathologic form of Aβ. No additive effect was found when the three NAEs were added together to the cell cultures (Benito et al., 2012).

Neuroprotective effects of NAEs were encountered in experimental models of *Parkinson disease (PD)*. Mice were treated with *1-methyl-4-phenyl-1,2,3,6-tetrahyropyridine (MPTP)*, which mimics biochemical and cellular changes that occur in idiopathic PD. Protection against MPTP-induced loss of *tyrosine hydroxylase (TH)* positive neurons in the substantia nigra was observed in mice treated with PEA (10 mg/kg). Chronic treatment was initiated 24 h after MPTP injection. Mice subjected to PEA treatment were also protected against the alterations of microtubule-associated protein 2a,b, dopamine transporter and nNos-positive cells in the substantia nigra. In addition, a reduction in microglial activation and the number of GFAP-positive astrocytes was observed, and MPTP-associated motor deficits were reversed. Furthermore, PEA was apparently protective against neurotoxicity and the ensuing functional deficits. This neuroprotective effect was exerted by activating PPARα (Esposito et al., 2012). Neuroprotective properties of OEA were also tested on experimental models of PD. OEA exerted cytoprotective effects both *in vitro* and *in vivo* models of *6-hydroxydopamine (6-OHDA)*-induced degeneration of substantia nigra dopaminergic neurons. OEA could exert a reduction in heme oxygenase-1 (oxidation marker) and OX6 (reactive microglia marker) *in vivo*, and protection against toxicity and cell death *in vitro*. However, these effects were U-shaped partial and dose-dependent, suggesting toxicity due to high drug concentration or an activation of opposing intracellular pathways by different OEA doses (Galan-Rodríguez et al., 2009). OEA-mediated neuroprotection of the nigrostriatal system was supported by a recent study. The *in vivo* model consisted of the intrastriatal infusion of 6-OHDA, which generates Parkinsonian symptoms. Neurotoxicity and behavioral deficits were less severe in the animals treated with the highest dose of OEA (5 mg/kg). In addition, 6-OHDA enhanced *heme oxygenase 1 (HO-1)* content was blocked by OEA (5 mg/kg). *In vitro*, OEA (0.5 and 1 μM) exerted significant neuroprotection on cultured nigral neurons via PPARα activation (González-Aparicio et al., 2014).

## Concluding Remarks and Perspectives

Glial cells are apparently responsible for producing NAEs (Walter et al., 2002; Muccioli and Stella, 2008), which constitute a homeostatic signaling system in response to brain damage and inflammation (Esposito and Cuzzocrea, 2013; Skaper et al., 2013). Accordingly, an augmentation of NAEs levels has been reported in brain aging (Pascual et al., 2014), Aβ-induced astrogliosis (Scuderi et al., 2011), neurodegeneration (Kallendrusch et al., 2010), SCI (Garcia-Ovejero et al., 2009), stroke (Schäbitz et al., 2002; Berger et al., 2004), and cerebral ischemia (Natarajan et al., 1986; Franklin et al., 2003). The Autacoid Local Injury Antagonism (Skaper et al., 1996) and the entourage effect (De Petrocellis et al., 2001; Cravatt and Lichtman, 2002; Ross, 2003; Bradshaw and Walker, 2005; Re et al., 2007; Liput et al., 2014) were proposed as mechanisms of NAEs homeostatic functions. However, in some pathological scenarios, NAEs endogenous synthesis is inadequate to control the inflammatory cascade (Skaper et al., 2013). When there is chronic non-resolving inflammation, dysfunctional reactive gliosis becomes detrimental and glial activity might not be beneficial any longer (Pekny and Pekna, 2015). Therefore, exogenous NAEs may exert neuroprotective effects by down-regulating astrocytes (Ahmad et al., 2012a,b; Scuderi et al., 2012, 2014), microglia (Galan-Rodríguez et al., 2009), or both microglia and astrocytes (Esposito et al., 2011, 2012). In this sense, glia constitutes a target for exogenous NAEs (Scuderi et al., 2013; Skaper et al., 2013). Moreover, inhibition of endogenous NAEs degradation could represent a complementary therapeutic approach for neuroinflammation. Potential selective *N-acylethanolamide-hydrolyzing acid amidase (NAAA)* inhibitors have shown anti-inflammatory effects (Solorzano et al., 2009; Saturnino et al., 2010; Li et al., 2012; Yamano et al., 2012). This fact supports the involvement of NAEs in the control of inflammation (Petrosino et al., 2010). However, future research should attempt to clarify the role of glial cells in brain injury, and their interaction with NAEs.

Neuroprotection exerted by exogenous PEA and OEA was observed in several *in vivo* and *in vitro* models of brain injury and neurodegenerative diseases (Genovese et al., 2008; Galan-Rodríguez et al., 2009; Esposito et al., 2011, 2012; Scuderi et al., 2011, 2012, 2014; Ahmad et al., 2012a,b; Benito et al., 2012; D’Agostino et al., 2012; Zhou et al., 2012; Mannelli et al., 2013; González-Aparicio et al., 2014; Sayd et al., 2015). However, the entourage effect was not referred as a mechanism of neuroprotection. In addition, a group of researchers has studied this issue in particular. PEA- mediated neuroprotective effects in dentate gyrus granule cells in excitotoxically lesioned OHSCs (Koch et al., 2011) were not exerted by AEA (Kreutz et al., 2007). Therefore, the entourage hypothesis could not explain PEA neuroprotective action, which was actually mediated by PPARα activation (Koch et al., 2011).

Peroxisome proliferator-activated receptor-α appears as a crucial site at which PEA and OEA generate their neuroprotective actions. Exogenous PEA and OEA have repeatedly exerted neuroprotective effects by activating the anti-inflammatory nuclear receptor PPARα (Lo Verme et al., 2005; Sun et al., 2007; Genovese et al., 2008; Scuderi et al., 2011, 2012, 2014; D’Agostino et al., 2012; Esposito et al., 2012; Zhou et al., 2012; Mannelli et al., 2013; González-Aparicio et al., 2014). Additionally, endogenous PEA and OEA have demonstrated protective effects via PPARα in activated cultured primary rat astrocytes subjected to Aβ accumulation (Scuderi et al., 2011) In fact, a recent hypothesis proposes that PPARs play a crucial role on metabolic and inflammatory compensation of astrogliosis and may interact with ligands on different metabolic pathways in order to supply energy to the neurons (Iglesias et al., 2016).

Moreover, some studies on neuroprotective effects of exogenous PEA and OEA revealed a co-occurrence between PPARα activation and down-regulation of glia (Esposito et al., 2012; Scuderi et al., 2012, 2014). Additionally, it was hypothesized that PEA-mediated PPARα activation could reduce significantly the number of microglial cells and counteract secondary neuronal damage of dentate gyrus granule cells in excitotoxically lesioned OHSCs (Koch et al., 2011). Further studies might help to elucidate whether NAEs-mediated PPARα activation is capable of counteracting glial activation.

## Author Contributions

All authors listed, have made substantial, direct and intellectual contribution to the work, and approved it for publication.

## Conflict of Interest Statement

The authors declare that the research was conducted in the absence of any commercial or financial relationships that could be construed as a potential conflict of interest.
